# Microbial Chemical Diversity and Cytotoxic Potential From Brazilian Ferruginous Caves: A Pioneering Metabolomic Survey

**DOI:** 10.1002/cbdv.202502912

**Published:** 2026-01-08

**Authors:** Natália Naomi Kato, Aline Figueiredo Cardoso, Bianca Del Bianco Sahm, Letícia Veras Costa‐Lotufo, José Augusto Pires Bitencourt, Norberto Peporine Lopes

**Affiliations:** ^1^ Núcleo de Pesquisa em Produtos Naturais e Sintéticos, Faculdade de Ciências Farmacêuticas De Ribeirão Preto Universidade De São Paulo (USP) Ribeirão Preto Brazil; ^2^ Instituto Tecnológico da Vale Desenvolvimento Sustentável Belém Brazil; ^3^ Laboratório de Farmacologia de Produtos Naturais Marinhos Instituto De Ciências Biomédicas, Universidade de São Paulo São Paulo Brazil

**Keywords:** extremophile, mass spectrometry, omics, subterranean, untargeted

## Abstract

Cave microbiomes represent a rich yet understudied source of chemical diversity with biotechnological properties. Microorganisms in these environments thrive under extreme conditions such as darkness, oligotrophy, and high concentrations of inorganic matter like iron ore. In this study, sediment samples were collected from the aphotic zone of a ferruginous cave in the National Forest of Carajás (Brazilian Amazon). Eight bacterial strains were isolated and taxonomically classified using 16S rRNA gene sequencing, revealing five genera: *Serratia*, *Bacillus*, *Enterococcus*, *Aneurinibacillus*, and *Comamonas*. Crude extracts from liquid cultures were analyzed using untargeted LC–MS/MS and processed through feature‐based molecular networking on the GNPS platform. The resulting network highlighted the production of structurally similar compound classes across different genera, including cyclopeptides, cholic acid derivatives, and indole alkaloids. Crude extracts were tested for cytotoxicity against HCT‐116 and 501mel tumor cell lines, with significant inhibition observed for extracts from *Aneurinibacillus*, *Comamonas*, and *Enterococcus*. Multivariate analysis linked cyclopeptide derivatives to cytotoxic activity. This study offers one of the first insights into the chemical potential of cave‐dwelling bacteria in Brazil and underscores their promise for future biotechnological exploration.

## Graphical abstract

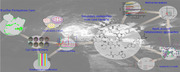

## 1 Introduction

Cave microbiomes remain largely unexplored in terms of microbial diversity and associated chemical interactions. Microorganisms play a fundamental ecological role in ferruginous cave environments by mediating iron cycling processes within the substrate, which contribute to the formation and stability of the cave rock structures. These microbial activities not only influence geochemical dynamics but also sustain the integrity of the cave ecosystem [1]. Brazil hosts some of the world's largest iron‐rich landscapes, where ferruginous caves represent a predominant speleological formation [2]. As one of the most widespread formations, pristine ferruginous caves host a remarkable and distinct microbial biodiversity that remains to be explored.

Several challenges hamper research in the cave extreme environments. Foremost among them is the difficulty of accessing pristine sampling areas, often located in the deep zones of caves [3, 4]. In addition, maintaining these microorganisms under controlled laboratory conditions poses major obstacles. The cultivability of cave bacteria is extremely low (estimated at approximately 0.02%), which limits monoculture‐based chemical studies [5]. These constraints highlight the urgent need for innovative approaches to better understand the microbial and chemical dynamics within cave systems.

Recent advances in microbiome research, particularly through high‐throughput techniques such as metagenomics and metabolomics, have significantly expanded our ability to probe microbial diversity and their chemistry. Metagenomics enables taxonomic classification via genetic sequencing [6], while metabolomics allows broad assessment of metabolic profiles [7, 8]. The integration of these techniques represents a powerful strategy for deciphering the complex ecology of cave microbiomes [9].

Prior work has demonstrated the biotechnological potential of cave‐dwelling microorganisms through the discovery of metabolites with antioxidant [10], cytotoxic [11, 12], and antibiotic properties [3, 13]. Here, we investigate the chemical diversity and the cytotoxic activity assay through metabolomics tools, such as annotation, molecular network clustering, and multivariate statistical analysis, to determine the active compounds with potential anticancer properties. These findings support the hypothesis that bacteria inhabiting ferruginous cave systems may represent a valuable source of novel bioactive compounds with therapeutic relevance.

## 2 Results and Discussion

### 2.1 Microbiological Diversity Recovered From the Ferruginous Cave

**FIGURE 1:**
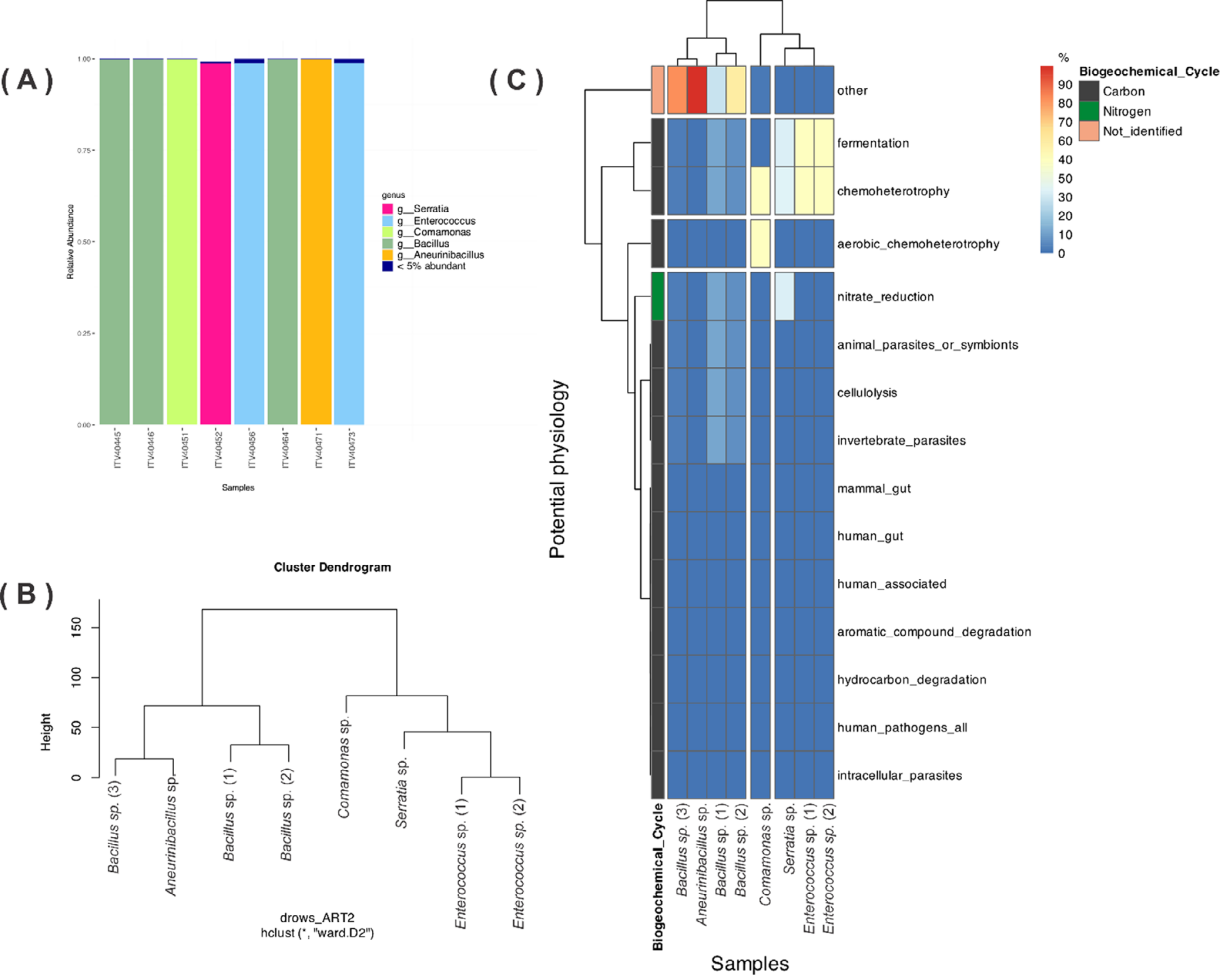
(A) Relative abundance of eight isolated bacteria at the genus level. (B) Hierarchical clustering analysis of taxonomic relationships among the recovered strains. (C) Heatmap with the predicted microbial metabolic pathways.

The investigation of the microbial diversity was conducted in the oligotrophic environment and the aphotic zone of the ferruginous cave. After collecting the sediment samples, eight bacterial strains were isolated based on their colony morphology, including characteristics such as color, elevation, form, and surface texture. Genomic sequencing of the 16S rRNA revealed that the isolated bacteria belong to five distinct genera: *Bacillus*, *Comamonas*, *Serratia*, *Enterococcus*, and *Aneurinibacillus*, shown in Figure 1A, and the clustering among the isolated is represented in the dendrogram of Figure 1B. While bacteria are ubiquitous in ecosystems, specific bacterial groups play crucial ecological roles in shaping the cave ecosystem [14].

The metabolic pathways were predicted for each isolated bacterium (Figure 1C), revealing their potential roles in the biogeochemical cycle. Among the eight strains, physiological pathway analysis indicated that most do not primarily rely on organic carbon as an energy source and exhibit relatively low aerobic activity. For *Serratia* sp., *Enterococcus* sp. (1), *Enterococcus* sp. (2), and *Comamonas* sp., the heatmap analysis suggests the active chemoheterotroph pathways and fermentation activity, particularly in Gram‐negative bacteria. This finding offers insight into the putatively active pathways in the isolated bacteria and underscores their ecological significance in modeling the environment in which they thrive.

According to Cardoso et al. [15], the presence of specific bacterial groups may be correlated with the iron cycle, whereas microorganisms participate in the dissolution and precipitation of the mineral in duricrust. Among the isolated bacteria, the *Bacillaceae* and *Paenibacillaceae* groups, both belonging to the *Bacillota* phylum, had already been reported in cave ecosystems [16, 17]. The *Bacillus* genus seems to possess resistance mechanisms to survive in oligotrophic substrates compared to other microbial groups [18] and also participates in geological formations by catalyzing reactions involving minerals [19]. In addition, the *Aneurinibacillus* genus had been isolated from soil samples [20] and, in particular habitats, in high temperatures and acidic conditions [21]. Thus, the cave environment is conducive to the isolation of *Bacillus* and *Aneurinibacillus* strains.

*Enterococcus* is an opportunistic bacterium commonly found in nutrient‐rich environments, serving as a fecal indicator, but it has been identified in extraenteric habitats [22]. Despite this bacterium being widely distributed in several substrates with organic availability and may be regarded as a pathogenic species, we considered it an indispensable group to understand the ecological and biotechnological function within the cave ecosystem.

The *Pseudomonadota* phylum is widely described in caves based on culture‐independent methodologies through sequencing of DNA fragments of metabarcoding analysis [23]. Also, these species participate in the carbon and nitrogen cycle and may be present from the entrance to the dark zones of the cave [14]. Despite the high diversity of *Pseudomonadota* species in caves correlating to the low amount of organic matter [24], we obtained two bacterial strains, *Serratia* sp. and *Comamonas* sp. Although these Gram‐negative bacteria are widespread in other substrates from the ecosystem, there is no investigation of the chemical profile of strains recovered from caves.

The species of the genus *Comamonas* are described in different niches as opportunistic bacteria [25]. Their main occurrence in nature is related to substrates with a high content of heavy metal pollutants, in which they participate in bioremediation processes [26] and are found in habitats with low nutrient availability, like caverns [24]. *Serratia* species, on the other hand, are reported in several substrates in caves from rich‐nutrient to the oligotrophic environment [27], and their presence was associated with the biomineralization process [27].

Considering the main bottlenecks to the cultivation of cave microorganisms, we move forward to study the chemical potential of the bacterial strains from a ferruginous cave in the Amazon rainforest and whether the particular environment could influence the production of metabolites with biological interest.

### 2.2 Chemical Profiling of Bacterial Strains

To further characterize possible chemical markers produced by bacteria isolated from iron‐rich substrates, the organic extracts were obtained from liquid cultures using ethyl acetate partition, which favored the recovery of compounds from medium to high polarities [11, 12, 17]. LC–MS/MS analysis detected 3672 features (MS1 and MS2) and was then used to build molecular networks, allowing us to observe the chemical profile of the different taxonomic groups (Figure S2), compound annotations by GNPS and other public databases in SIRIUS.

The molecular network showed a high diversity of features grouped according to spectral similarity (Figure S2). After the processing in the FBMN, we followed up using microbeMASST, as a search tool from the GNPS2 platform, in order to correlate the occurrence of the consensus spectra in the microbial monocultures and performed enrichment analysis [28]. The main specialized annotated metabolites belong to cyclopeptides, dipeptides, diketopiperazine alkaloids, and cholane steroids classes. After the visualization of the features annotation and the consensus spectral molecular network, we move forward with the dereplication of the compounds, considering the occurrence of the metabolites in the bacterial groups and the fragmentation patterns of precursor ions.

**FIGURE 2:**
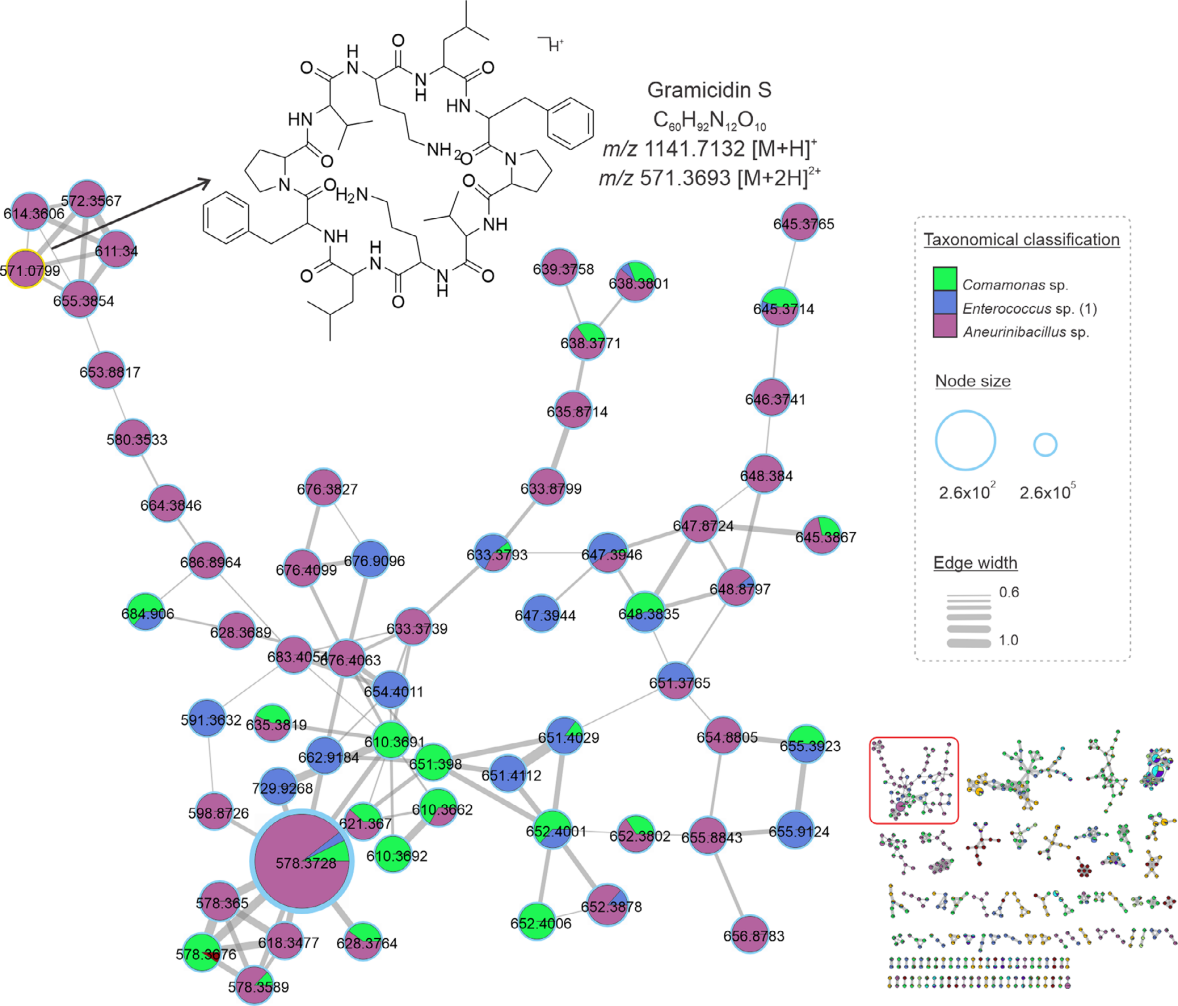
Cluster from *Aneurinibacillus*, *Enterococcus*, and *Bacillus* crude extracts. The consensus spectra refer to the cyclopeptides class, putatively determined as the Gramicidin S analogs. The genera are represented in each color, which is described in the legend, and the node size corresponds to the relative abundance of ions. The edge size represents the cosine score among the nodes.

Herein, cyclopeptides were found in extracts from *Comamonas* sp., *Enterococcus* sp. (1), and *Aneurinibacillus* sp. the molecular network in Figure 2 represents the clustering of consensus spectra of cyclopeptides. This class of substances is known to be widely produced by *Aneurinibacillus* species [29]. Gramicidin S was the compound annotated in this cluster by GNPS, which corresponds to *m*/*z* 1141.7132 [M+H]^+^ C_60_H_93_N_12_O_10_^+^. Based on this annotation and the occurrence of the Gramicidin derivatives in the *Aneurinibacillus*, we proposed analogs according to the cluster of the consensus spectra and fragmentation patterns. The most intense peak in the chromatogram corresponds to the ion *m*/*z* 1155.7244 [M+H]^+^, C_61_H_95_N_12_O_10_^+^, which we putatively determined as the analog to Gramicidin S containing a leucine unit instead of valine (Figure 2).

**FIGURE 3:**
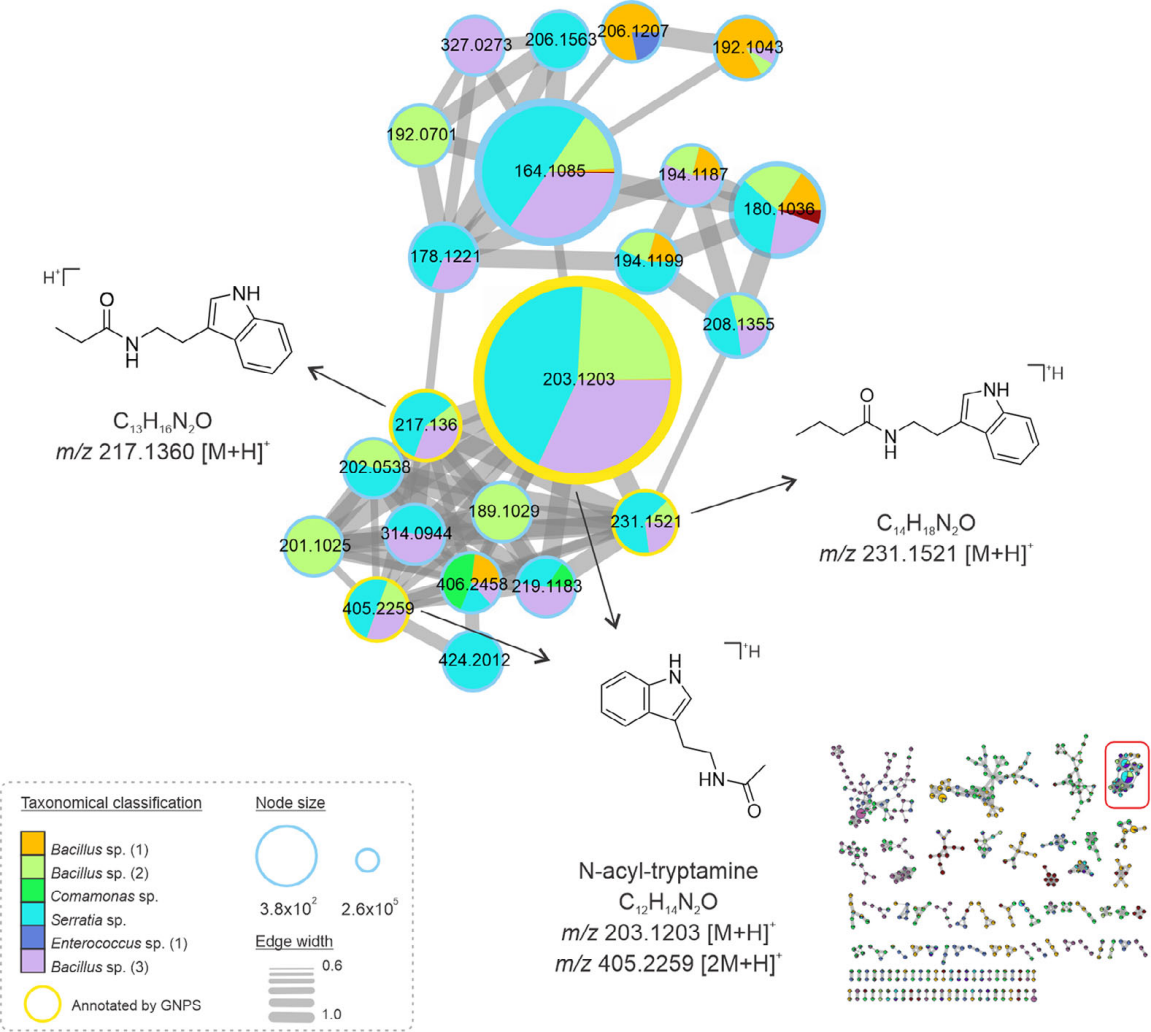
Cluster of the consensus spectra of tryptamine‐like derivatives. Strains are represented by color, and node size corresponds to the relative abundance of ions. Nodes with yellow borders were annotated by the GNPS library. The edge size represents the cosine score among the nodes.

Other metabolites from the tryptophan biosynthetic pathway were detected in *Bacillus*, *Comamonas*, *Serratia*, and *Enterococcus* extracts, from which tryptamine analogs were annotated with different side chains coupled to the indole ring (Figure 3). One of the most intense peaks is an N‐acyl‐tryptamine, detected in the protonated and dimer forms: corresponding to *m*/*z* 203.1203 [M+H]^+^ and *m*/*z* 405.2259 [2M+H]^+^, respectively. The detected analogs exhibited the N‐acyl moiety, but with variations solely in the number of carbon atoms attached to the side chain, corresponding to the ions *m*/*z* 217.1360 [M+H]^+^ and *m*/*z* 231.1521 [M+H]^+^, distinguished by a difference of 14 units (R‐CH_2_‐R). Interestingly, these compounds had been reported to occur in two species of *Bacillus*, isolated from soil samples, and presented antibacterial and antifungal effects [30]. Moreover, the synthesis of molecules inspired by tryptamine‐derived Schiff bases has shown potent pharmacological activities, including antibacterial and cytotoxic effects on tumoral cell lines [31]. Thus, we hypothesize that the *Bacillus* sp., *Comamonas* sp., *Serratia* sp., and *Enterococcus* sp. strains recovered in this work, which are tryptamine‐like compounds producers, could be potential sources of bioactive compounds for distinct pharmacological interests.

**FIGURE 4:**
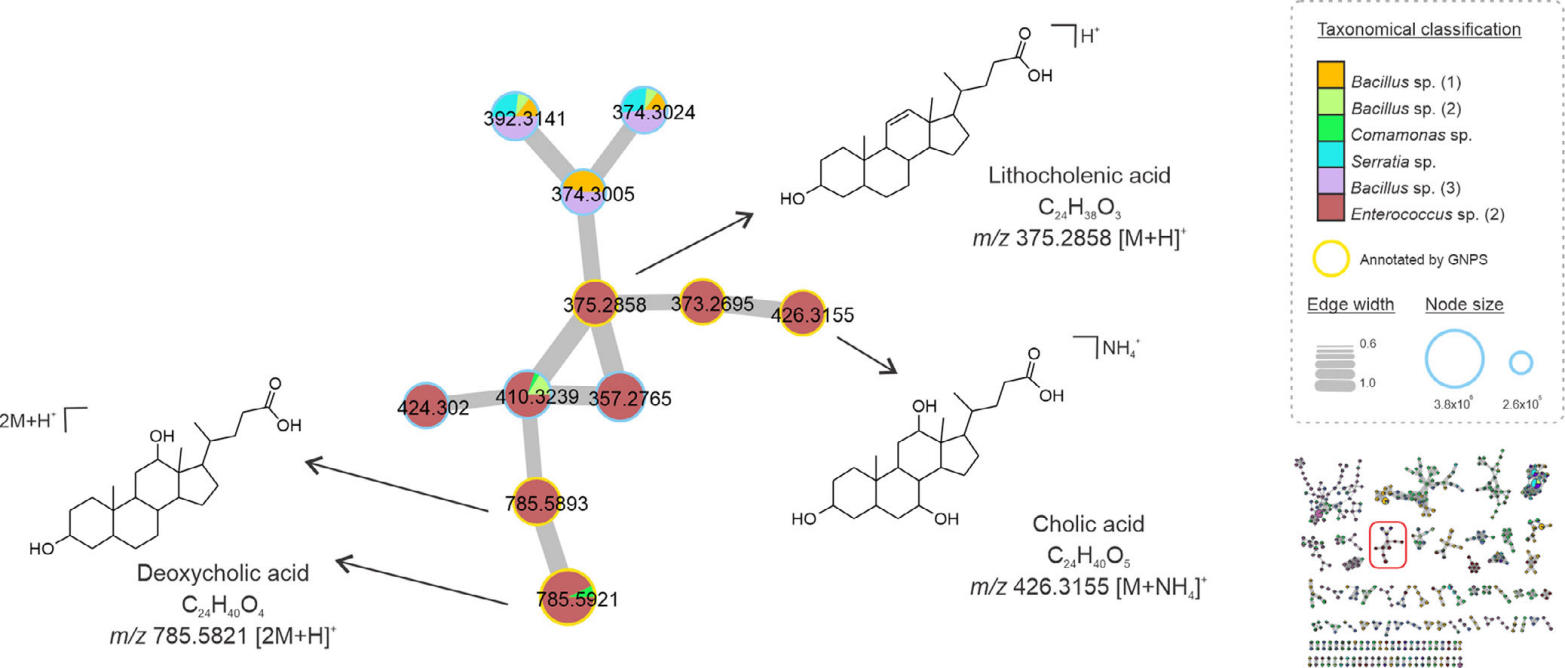
Consensus spectra of cholic acid derivatives from genera *Bacillus*, *Comamonas*, *Serratia*, and *Enterococcus*. Strains are represented by color, and node size corresponds to the relative abundance of ions. Nodes with yellow borders were annotated by the GNPS library. The edge size represents the cosine score among the nodes.

Cholic acid derivatives were also annotated in our molecular network (Figure 4). Although this class of compounds is widely produced by eukaryotic cells, the biosynthesis of sterols by prokaryotes is well understood, but it has been described among methanotrophic bacteria [32]. The same was found in strains isolated from a freshwater lake [33] and from the marine environment [34]. Here, cholic acid (*m*/*z* 426.3155 [M+NH_4_]^+^) and derivatives were annotated in the crude extracts of *Enterococcus*, *Bacillus*, and *Serratia* gender specimens. Derivatives were detected and annotated in the reduced form of lithocholic acid (*m*/*z* 375.2858 [M+H]^+^, C_24_H_38_O_3_), the dimers of deoxycholic acid (*m*/*z* 785.5821 [2M+H]^+^, C_24_H_40_O_4_), and other analogs in the cluster were annotated by GNPS. In addition, we searched the spectral data in the microbeMASST database, showing that these derivatives are found in various microbial groups (Figure S3). In general, several factors can influence bacterial metabolic pathways, resulting in the production of the same or similar compound classes despite different genotypes, including adaptation, competition, and communication abilities [35].

Despite being a very useful tool for metabolomics studies, the GNPS platform and its database is dependent on being replenished by its users, so that more spectra can be replicated and their compounds annotated [36]. Furthermore, the cave environment remains obscure within the universe of natural products, especially regarding the yet undiscovered biodiversity of microorganisms existing in Brazilian caves. With the aid of emergency tools in metabolomics of microbial search, such as the microbeMASST [28], we believe that new spectral data from microorganisms will soon be available, enabling increased annotations of compounds derived from cave‐dwelling bacteria as well. In this sense, our work presents a rich contribution both to the repository of platform databases and also to the understanding of the chemical and biological diversity in bacteria recovered from a ferruginous cave in the Brazilian Amazon region. This is an important step in expanding the knowledge and facilitating research on cave microbiomes.

**FIGURE 5:**
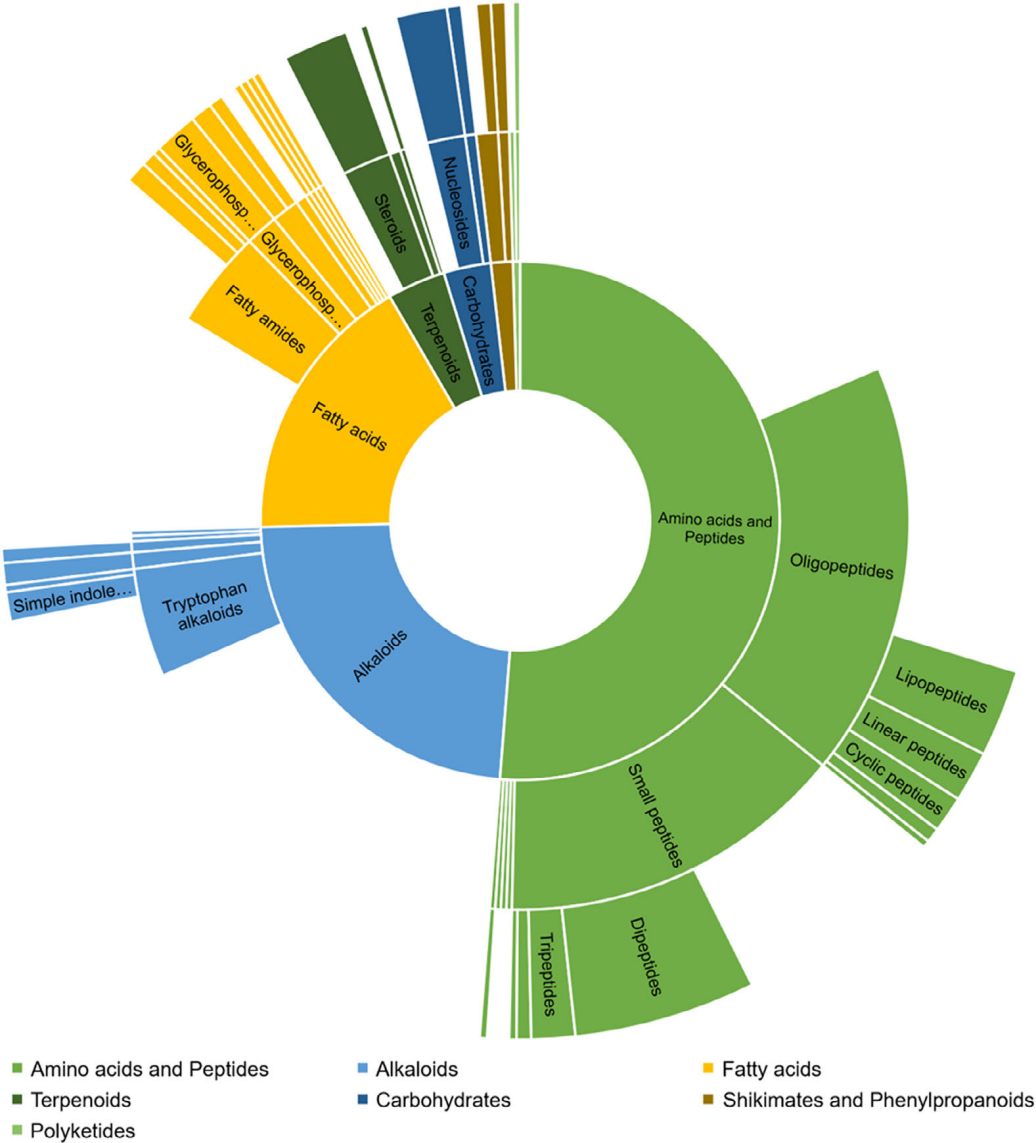
Annotation of features and prediction of biosynthesis pathway by SIRIUS. Each feature was sorted according to the biosynthesis pathway (inner ring), superclasses (middle ring), and classes (outer ring) from NPClassifier. Sunburst plots are built considering 70% of probability to the pathway, superclass, and class.

SIRUS metabolomics integrates several tools to extract chemical information from detected features, considering the high‐resolution *m*/*z* values with their MS/MS spectra; thus, 674 features were classified according to the Natural Products Classes represented in the Sunbrust Plot of Figure 5. The software provides molecular formula predictions, candidate chemical structures, compound classification based on NPClassifier or Classyfire, and pathway annotation [37]. The prediction of biosynthetic pathways is based on the class‐level annotation and does not consider the bacterial biosynthesis of specialized metabolites, PKS and NPRS pathways. The main features were classified within amino acids and peptides pathways, including the alkaloids that are probably produced from essential amino acids. The detection of Shikimate and Phenylpropanoids corresponds to some features of the culture medium. Nevertheless, this classification highlights the remarkable diversity of metabolites produced by cave‐dwelling bacteria.

### 2.3 Screening of Cytotoxic Potential on Tumoral Cell Lines

To check for the biomedical potential of chemicals produced by isolated bacteria, the crude extracts were tested for their cytotoxicity activity against two tumoral cell lines: 501mel (metastatic melanoma) and HCT‐116 (colorectal carcinoma), in addition to the NIH‐3T3 fibroblast cell line as the non‐cancerous model. For the assay, we used concentrations of 50 and 5 µg/mL for each extract, and the cell growth inhibition (%) was checked after 72 h of incubation by MTT assay. We consider active extracts that inhibit at least 70% of cell growth.

Considering the standard deviation, our results showed the cytotoxicity potential of *Enterococcus* sp. (1) and *Aneurinibacillus* sp. extracts, in the highest concentration, both tumoral cell lines, while *Aneurinibacillus* sp. achieved almost 100% inhibition in all lineages, including normal fibroblasts (Figure 5; Table S2). This finding demonstrates that *Aneurinibacillus* sp. extract has low specificity toward tumor cells, and that, in this scenario, the extracts of *Enterococcus* sp. (1) and *Comamonas* sp. would be more suitable to be explored in terms of cancer treatment due to its selective effectiveness against tumor cell lines.

Given that the viability assay is an initial screening and a critical step in identifying potential antitumor activity, further in‐depth investigation is required to elucidate the mechanism of action and to accurately assess toxicity in both normal and cancerous cells. The HCT‐116 and 501mel cell lines are characterized by aggressiveness [38] and metastatic properties, respectively; even so, the crude extracts already exhibited antiproliferative effects, making them strong candidates for bio‐guided studies of fractions and isolated compounds. Therefore, additional studies are necessary to further evaluate these extracts as potential therapeutic agents.

Cave‐dwelling bacteria have been explored for their anticancer potential, demonstrating promising cytotoxic effects in vitro across different tumoral cell lines such as colon [9], lung, cervix, and ovary carcinomas [12, 39] and skin cancer [40]. However, it is worth noting that Gram‐positive actinobacteria (phylum Actinomycetota) are the most explored group in caves, both due to their abundant occurrence in this environment and also due to their ability to biosynthesize specialized secondary metabolites [9, 41]. Most bioprospecting work conducted with cave microorganisms has focused on this group, which has revealed the isolation of cytotoxicity compounds such as hypogeamicins [12] and huanglongmycin [40]. In the present work, however, we present the cytotoxic potential of Gram‐negative bacteria, possibly opportunistic, revealing that other groups existing in the cave‐dwelling environment have the potential for biotechnological exploration.

Notably, the extracts of *Enterococcus* sp. (1) and *Aneurinibacillus* sp. contain substances with inhibitory action on the tested cells, and efforts should be made to isolate and characterize the compounds responsible for their activity. Once again, it should be noted that the exploration of cave environments in Brazil may be a great source to reveal new chemical entities, which could lead to the development of innovative therapies for the treatment of cancer and other emerging diseases.

### 2.4 Correlation Between Biological Activity and Chemical Profiling

We apply multivariate analysis aiming at the evaluation of each bacterial strain according to the metabolome for the characterization of discriminant compounds. The principal component analysis (PCA) provided, as an unsupervised method, the good separation of the bacterial samples, the quality control, and blanks, and was validated according to *R* ^2^ and *Q* ^2^ (Figure S4).

**FIGURE 6:**
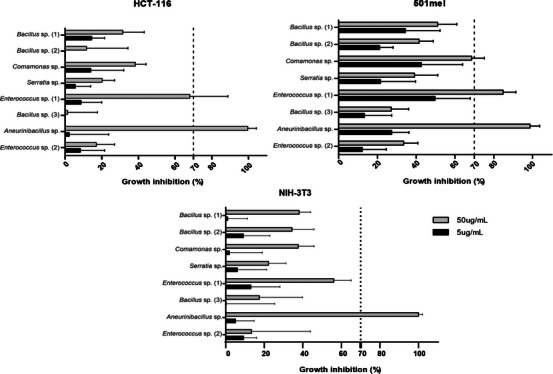
Cytotoxic potential of bacterial crude extracts against tumoral cell lines HCT‐116, 501mel, and NIH‐3T3. Extracts were tested in concentrations of 5 µg/mL (black bars) and 50 µg/mL (gray bars), which represents the mean percentage of cell growth inhibition from three independent experiments. The standard deviation is represented by thin bars.

After the PCA model validation, we proceed with the supervised analysis using the partial least squares–discriminant analysis (PLS‐DA) based on the cytotoxic activity, shown in Figure 6, in which extracts were categorized as active or inactive. The *R* ^2^ *X*, *R* ^2^ *Y*, and *Q* ^2^ showed 0.67, 0.998, and 0.879, respectively, which means good fitness and robustness of the analysis. The next step was the determination of the discriminant features in the active extracts.

**FIGURE 7:**
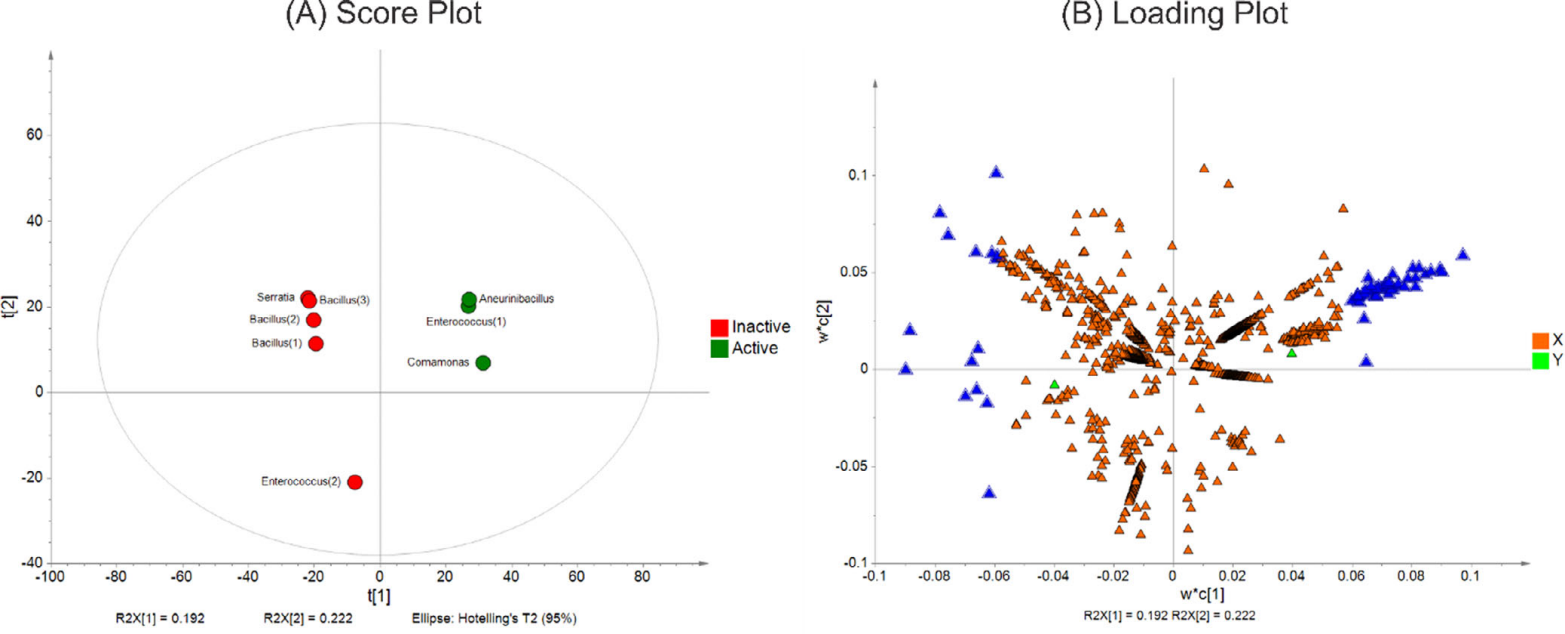
(A) Score plot of the bacterial samples, the active samples are in green, and the inactive samples are in red. (B) Loading plot of MS1 features, the blue triangle represents the discriminant features, and the green box represents the active and inactive biological effects, which enables the correlation between features and cytotoxic assay.

By separating active and non‐active extracts, PLS‐DA allows the identification of discriminant features responsible for the differentiation between the groups (Figure 7B, blue triangle). Based on this, we identified cyclopeptide derivatives from the *Aneurinibacillus*, *Comamonas*, and *Enterococcus* (1) extracts (Figure 2) as the features from the active extracts, since their VIP score was above 2 (Table S3). This finding enables the prediction of the compounds responsible for biological activity with pharmacological interest. Metabolomics approaches facilitate the connection between chemical data and biological properties to discover active compounds, thus uncovering insights into poorly studied ecosystems.

Among the three active extracts, there are biological properties correlated with their ecological role in the different ecosystems, but not with their cytotoxic effects on cancer cell lines. The Gramicidin S cyclopeptides from *Aneurinibacillus* sp. genus shows potential as a biocontrol agent against pathogens in plants when isolated from soil‐borne sources [29]. Although the *Comamonas* sp. and *Enteroccus* sp. genera are well‐known for their roles in bioremediation process, and are also pathogenic [22, 24]; their capacity to produce secondary metabolites suggests they can provide scaffolds for therapeutic agents. Since the natural cyclic peptides are widely associated with anticancer properties [42], our results support the effectiveness of Gramicidin S‐type cyclopeptides and analogs against tumor cell lines.

## 3 Conclusions

This study provides a pioneering chemical investigation of microbial strains isolated from a ferruginous cave in the Brazilian Amazon. We identified five bacterial genera capable of producing diverse metabolites, including cyclopeptides, tryptamine analogs, and cholic acid derivatives, with some extracts displaying promising cytotoxic activity. The overlap of compound classes among phylogenetically distinct genera suggests that cave‐specific selective pressures may shape convergent metabolic traits. Our findings contribute not only to the limited global knowledge of cave microbiomes but also stand among the first chemical studies of subterranean Brazilian ecosystems. By integrating untargeted metabolomics and multivariate statistics, we establish a workflow that highlights cave‐dwelling bacteria as valuable targets for natural product discovery. This work opens new avenues for exploring the unique biosynthetic potential of Brazil's extensive cave systems and underscores their relevance for biotechnological and pharmacological innovation.

## 4 Experimental

### 4.1 Cave Description and Sample Collection

**FIGURE 8:**
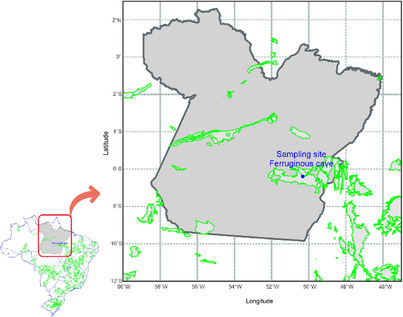
Map of Brazil with the cave occurrence areas (outlined in green). Blue circle corresponds to the collection site in the ferruginous cave's complexity located in Pará state. Adapted from Centro Nacional de Pesquisa e Conservação de Cavernas—CECAV (ICMBIO, 2023).

Sediment samples were collected in the dark zone from a ferruginous cave located at Canaã do Carajás, PA, Brazil (6°23′48.296″ S, 50°19′24.989″ W). This region is characterized by extensive iron‐rich landscapes called Carajás canga, encompassing the ferruginous caves (Figure 8). The cave exhibited localized dripping and a consistently wet floor throughout its extension. An amount of approximately 1 g of sediment was collected aseptically, using spatulas and Falcon tubes, and cooled at 4°C until taken to the laboratory to perform the microbiology and genomic protocols. The collection of the samples was authorized and registered by Brazilian agencies, such as Sistema de Autorização e Informação em Biodiversidade (SISBIO 79860‐2) and Sistema Nacional de Gestão do Patrimônio Genético e do Conhecimento Tradicional Associado (SisGen AD421A0).

### 4.2 Cultivation and Isolation of Cave Bacterial Strains

The cave sediment samples were subjected to a 10‐fold serial dilution in sterile saline solution (0.85% NaCl) under aseptic conditions in a laminar flow cabinet. Subsequently, 100 µL aliquots from appropriate dilutions were inoculated, in a proportion of 1:10, onto tryptic soy agar (TSA) plates. The culture medium was composed of pancreatic digest of casein (17 g/L), papaic digest of soybean meal (3 g/L), yeast extract (6 g/L), dextrose (2.5 g/L), sodium chloride (5 g/L), and agar (6 g/L), all sourced from Sigma (Sigma‐Aldrich), and adjusted to pH 7.3. The medium was autoclaved at 121°C for 20 min before use. Plates were incubated at 24°C in the dark, and based on the growth and morphological characteristics of the colonies, individual bacterial strains were isolated for subsequent genome sequencing and chemical profiling. The isolated bacterial strains were transferred into 100 mL of tryptic soy broth (TSB) culture media (same composition of TSA but no agar) and incubated for 7 days at 24°C under agitation at 180 rpm. One part of the liquid media was separated to perform the genomic sequencing, and the other to the extraction of crude extracts for chemical analysis.

### 4.3 Taxonomical Profiling

DNA from isolated bacterial cultures was extracted in triplicate after filtering 15 mL of each sample through sterile 0.22 µm membranes (Millipore). The filters were cut into small pieces with a sterile scalpel, and DNA was extracted using the PowerSoil DNA Isolation Kit (QIAGEN) following the manufacturer's instructions. DNA concentration and integrity were evaluated using a Qubit 3.0 fluorometer (Thermo Fisher Scientific) and 1% agarose gel electrophoresis, respectively. PCR amplification targeted the V3–V4 region of the 16S rRNA gene using universal primers S‐D‐Bact‐0341‐b‐S‐17 and S‐D‐Bact‐0785‐a‐A‐21 [15, 43]. Reactions were performed in a 25 µL mix and amplified using the protocol described by Cardoso et al. [15].

Amplicon libraries were prepared following Illumina's 16S Metagenomic Sequencing Library Preparation protocol. Indexing was performed using KAPA HiFi HotStart ReadyMix (Sigma‐Aldrich), and the resulting libraries were purified with AMPure XP beads (Beckman Coulter). Library quality and fragment size distribution were assessed on a Bioanalyzer 2100 (Agilent), and all samples were normalized to 4 nM before sequencing on an Illumina MiSeq platform using the MiSeq Reagent Kit v3 (600 cycles). This approach provided high‐throughput, reproducible, and genus‐level taxonomic resolution across all cultivated isolates, consistent with the ecological and metabolomic objectives of this study. Sequence processing followed the PIMBA pipeline [44], including quality filtering with Prinseq, OTU clustering at 97% similarity using VSEARCH, chimera removal, and taxonomic assignment against the SILVA SSU rRNA database (release 132).

The prediction of the microbial metabolic pathway was performed using the functional annotation of prokaryotic taxa (FAPROTAX, version 1.2.4). The database provides the metabolic functions of bacteria OTUs through 16S rRNA gene sequencing [45].

### 4.4 Crude Extract Preparation

After growing in the TSB media, inoculums and control were extracted by liquid–liquid partition using organic solvent ethyl acetate—1:1 (Labsynth, P.A.‐A.C.S 100%) and dried under reduced pressure at 35°C. One part of the extracts was used to perform the cytotoxic activities in vitro, and the other part was used for LC–MS/MS chemical analysis.

### 4.5 Data Acquisition by Using LC–MS/MS

The crude extracts were solubilized in a concentration of 1.5 mg/mL in methanol:water (1:1) and filtered using and 0.22 µm and 3 mm PTFE filter (Millex, Millipore). Each sample was pooled as a quality control. A volume of 3 µL of each sample was analyzed using a high‐performance liquid chromatography (HPLC), Shimadzu, with two bombs LC‐20AD, SIL‐20A auto‐injector, diode array detector (DAD) SPD‐M20A, CBM‐20A controller, and CTO‐20A oven coupled to mass spectrometry microTOFIII (Bruker Daltonics) with electrospray ionization followed by quadrupole and time‐of‐flight (Q‐TOF) mass analyzer. The chromatographic separation was performed using a Kinetex Core Shell C18 column, 2.6 μ, 100 × 4.6 mm, and the mobile phase was (A) Water with 0.1% formic acid and (B) Acetonitrile with 0.1% formic acid. The elution gradient method was: 0–0.01 min—10%B, 0.01–25 min—100%B, 25–27 min—100%B, 27–28 min—10%B, 28–30 min—10%B, with a total flow of 0.35 mL/min, and oven temperature of 40°C. The MS data were acquired following the parameters: 4000 V of capillarity voltage, 200°C dry heater, 9 L/min of dry gas, the nebulizer at 4.4 bar, and the target mass from 100 to 1200 *m*/*z*. To perform the fragmentation of selected precursor ions, the collision energy was set to 10 eV.

### 4.6 Data Pre‐Processing, Processing, and Chemical Profiling

After the acquisition of the LC–MS/MS spectral data, we converted them to .mzXML, using MSConvert. We preprocessed the spectral data following the feature‐based molecular network (FMBN) workflow, which allows the separation of isomers according to the retention time and alignment across the samples. Thus, the converted data were submitted to the MZmine 3.9.0 to perform the mass detection, chromatogram building, spectral deconvolution, isotopic grouping, and join alignment; the values of set parameters were in Table S1.

We submitted to the Global Natural Products Molecular Network (https://gnps2.org/homepage), an open‐source and online platform to analyze the consensus spectra clustering and compound annotation. The parameters used in the GNPS2 were precursor ion tolerance 0.03 Da and fragment ion tolerance 0.03 Da; minimum cosine 0.6 and minimum matched peaks 4; library minimum cosine 0.6 and library minimum matched peaks 4, Top‐K of 1, and no normalization. The features from the solvent blank and the culture media components were removed. In the same environment, we performed the analysis in the microbeMASST, a metabolomics search tool, to check the occurrence of the consensus spectra in the microbial database. Aiming to improve the reliability of annotated compounds and their analogs, we performed the dereplication of metabolites considering the *m*/*z* ratio in high resolution with an error up to 10 ppm, mass fragmentation patterns, and UV wavelength. The molecular networks were visualized and analyzed in Cytoscape 3.10.2 (Institute for Systems Biology, Seattle, WA, USA).

The preprocessing spectral file was analyzed using SIRIUS software (version 6.1.1). Based on the high‐resolution *m*/*z* values and fragmentation patterns, SIRIUS provides the molecular formula (Denovo + bottom up) considering the [M+H]^+^, [M+K]^+^, [M+Na]^+^ adducts, as well as compound class and biosynthesis pathways prediction through CANOPUS and annotation using public database (e.g., PubChem).

### 4.7 Cytotoxicity Evaluation in Tumor Cell Lines

Crude extracts were solubilized in 0.2 µm sterile‐filtered dimethyl sulfoxide (DMSO—Labsynth, P.A.‐A.C.S 100%) at 10 and 1 mg/mL stock solutions. For cytotoxicity bioassay, we selected two tumoral cell lines and a normal one (noncancerous): HCT‐116 (human colon carcinoma cell line, RRID: CVCL_0291, from Cell Bank from Rio de Janeiro/BCRJ) and 501mel (human melanoma cell line, RRID:CVCL_4633‐ATCC), which were cultivated in Gibco RPMI 1640 culture media (Thermo Fisher Scientific), and NIH‐3T3 (Murine Fibroblast Cell Line, RRID: CVCL_0594, from Cell Bank from Rio de Janeiro/BCRJ) which was cultivated in Gibco DMEM‐high glucose culture media (Thermo Fisher Scientific). Both culture media were supplemented with 10% bovine fetal serum (Vitrocell) and 1% antibiotics (penicillin 10 000 U/mL, streptomycin 10 µg/mL, and amphotericin B 25 µg/mL—Thermo Fisher Scientific). For experiments, 1 × 10^4^ cells/well were seeded in 96‐well cell culture plates and, after 24 h, treated with crude extract samples at final concentrations of 5 µM and 50 µM, in duplicates. Doxorubicin was used as a positive control, and DMSO as a vehicle‐negative control. After 69 h of incubation in a controlled atmosphere with 5% CO_2_ and a temperature of 37°C, the supernatant was replaced by a culture medium containing 0.5 mg/mL methyl‐thiazolyl‐tetrazolium (MTT—Sigma‐Aldrich), followed by re‐incubation under the same conditions. Three hours later, the supernatant was removed until completely dried, and the violet precipitated formazan crystals were dissolved in 150 µL of DMSO for absorbance measurement at 570 nm using a BioTek Synergy HTX Multimode Reader [46]. The percentage of cell growth inhibition for the samples was determined in GraphPad Prism v10.0.

### 4.8 Chemometrics Analysis

The quantitative table containing the features and the respective peak areas was used to perform the chemometrics analysis, in which the areas were normalized by Log10 transformation. After this, the data were submitted to the statistical analysis, through SIMCA 13.0.3 software, and we obtained the PCA and the PLS‐DA with cross‐validation using the *Q* ^2^ and *R* ^2^ values. The supervised analysis allows the discrimination of the features with VIP above 2 that correlate with the biological activity, showing a prediction of metabolites responsible for the cytotoxic properties.

## Author Contributions

**Natália N. Kato**: conceptualization, methodology, investigation, data curation, formal analysis, writing – original draft preparation, writing – review and editing. **Aline F. Cardoso**: investigation, methodology, and formal analysis. **Bianca B. Sahm**: investigation, methodology, formal analysis, and writing – review and editing. **Letícia V. Costa‐Lotufo**: investigation, funding acquisition, writing – review and editing. **José Augusto P. Bitencourt**: investigation, data curation, funding acquisition, writing – original draft preparation, writing – review and editing. **Norberto P. Lopes**: conceptualization, investigation, funding acquisition, supervision, writing – original draft preparation, writing – review and editing.

## Conflicts of Interest

The authors declare no conflicts of interest.

## Supporting information




**Supporting File 1**: cbdv70822‐sup‐0001‐SuppMat.docx

## Data Availability

The data that support the findings of this study are available in the Supporting Information of this article.
