# Supplementary material for: Microbial Chemical Diversity and Cytotoxic Potential From Brazilian Ferruginous Caves: A Pioneering Metabolomic Survey
Source: Chem Biodivers. 2026 Jan 8;23(1):e02912. doi: 10.1002/cbdv.202502912 (PMC12781163; doi:10.1002/cbdv.202502912)
Supplement: Supplementary file 1 — Supporting File 1: cbdv70822‐sup‐0001‐SuppMat.docx [file CBDV-23-e02912-s001.docx]

**Microbial chemical diversity and cytotoxic potential from Brazilian ferruginous caves: a pioneering metabolomic survey**

Natália Naomi Kato^1^, Aline Figueiredo Cardoso^2^, Bianca Del Bianco Sahm^1,3^, Letícia Veras Costa-Lotufo^3^, José Augusto Pires Bitencourt^2^, Norberto Peporine Lopes^1, *^

^1^Núcleo de Pesquisa em Produtos Naturais e Sintéticos, Faculdade de Ciências Farmacêuticas de Ribeirão Preto, Universidade de São Paulo (USP), Av. do Café s/n°, Ribeirão Preto - SP 14040-903.

^2^Instituto Tecnológico da Vale Desenvolvimento Sustentável, R. Boaventura da Silva, 955, Belém - PA, 66055-090.

^3^Laboratório de Farmacologia de Produtos Naturais Marinhos, Instituto de Ciências Biomédicas, Universidade de São Paulo, Av. Prof. Lineu Prestes 1524, São Paulo - SP, 05508-000

**
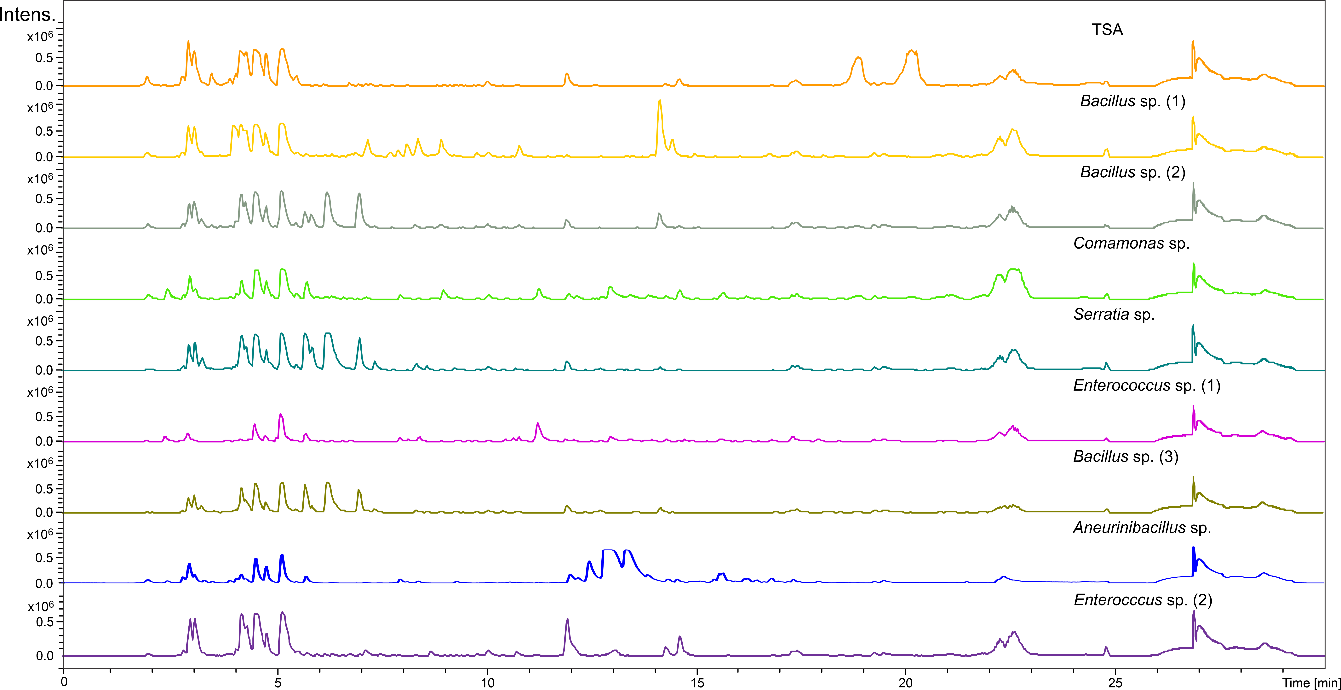
**

**Figure S1:** Chromatograms from LC-MS/MS analysis of eight isolated bacteria strain.

| Table S1: Parameters used in the preprocessing in MZmine 3.9.0 | | |
| --- | --- | --- |
| Mass detection | MS1 | 1E3 (centroid) |
|  | MS2 | 1E2 (centroid) |
|  | Min. cons. scan | 5 |
| ADAP Chromatogram Build | Min. intens. for cons. scans | 1E3 |
|  | Min. absolute height | 1E3 |
|  | m/z tolerance | 0.02 |
| Spectral deconvolution | MS/MS pairing MS1 to MS2 (m/z) | 0.03 |
|  | RT tolerance (min.) | 0.2 |
|  | S/N threshold | 10 |
|  | Min. abs. height | 1E3 |
|  | Coeff./area threshold | 10 |
|  | Peak duration range | 0.2 to 2 |
|  | RT wav. range | 0.01 to 0.1 |
| Isotopic grouping | m/z tolerance (intra-sample) | 0 .02 |
|  | RT tolerance | 0.02 (abs./min.) |
|  | Max. Charge | 2 |
| Alignment | *m/z* tolerance | 0.05 |
|  | weight for *m/z* | 70 |
|  | weight for RT | 30 |
|  | RT tolerance | 0.3 |

**
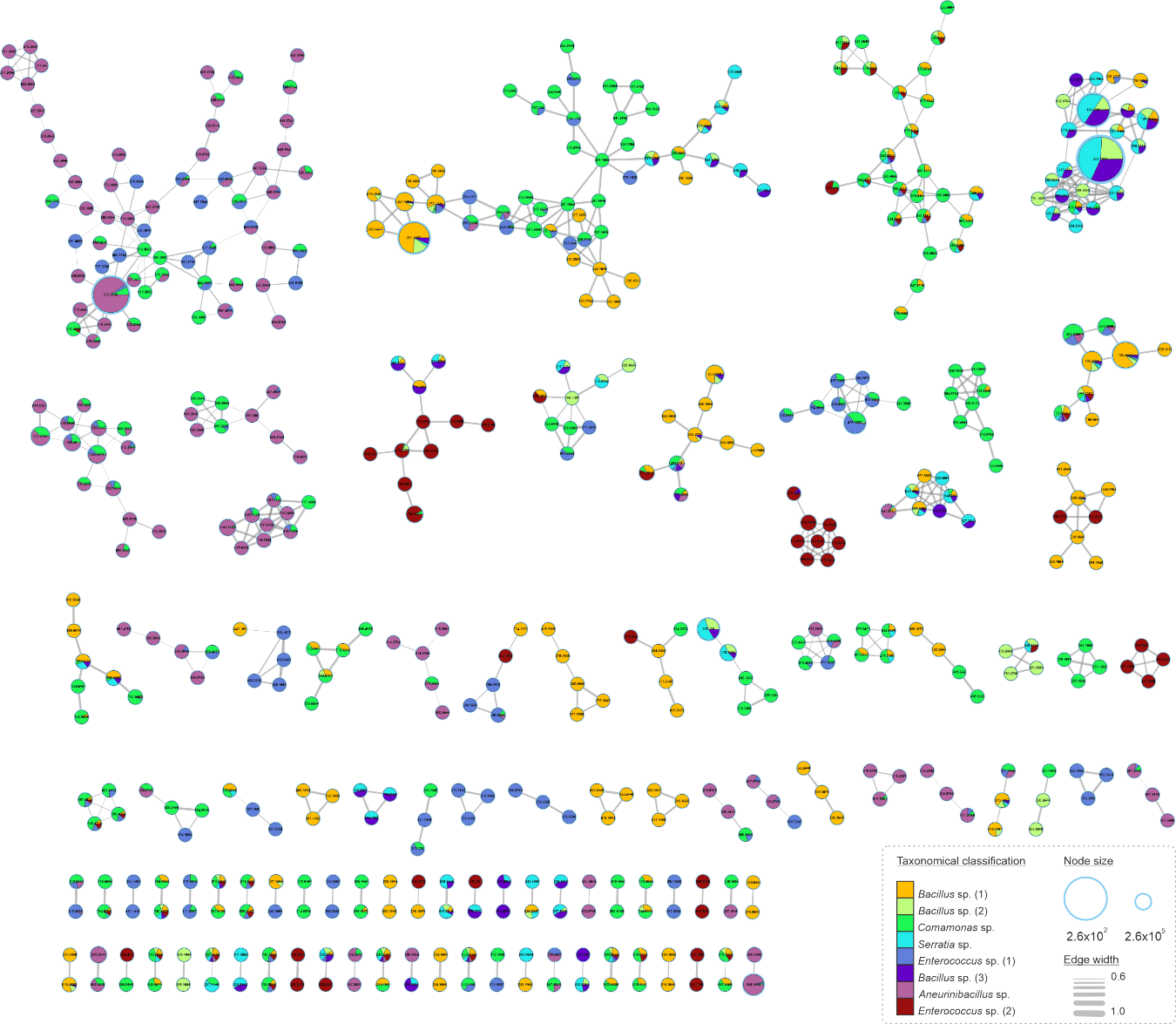
**

**Figure S2:** Molecular network of all clusters obtained by MS/MS analysis in positive ionization mode. Each color represents an isolated bacterium, whose genera are described in the legend and the node size corresponds to the relative abundance of ions. The edge size represents the cosine score among the nodes.

**Data availability:** The Feature Based Molecular Network workflow is available in the GNPS2 platform. The data can be assessed by the following link: https://gnps2.org/status?task=70628d36df1a461c983a3aba633d4612


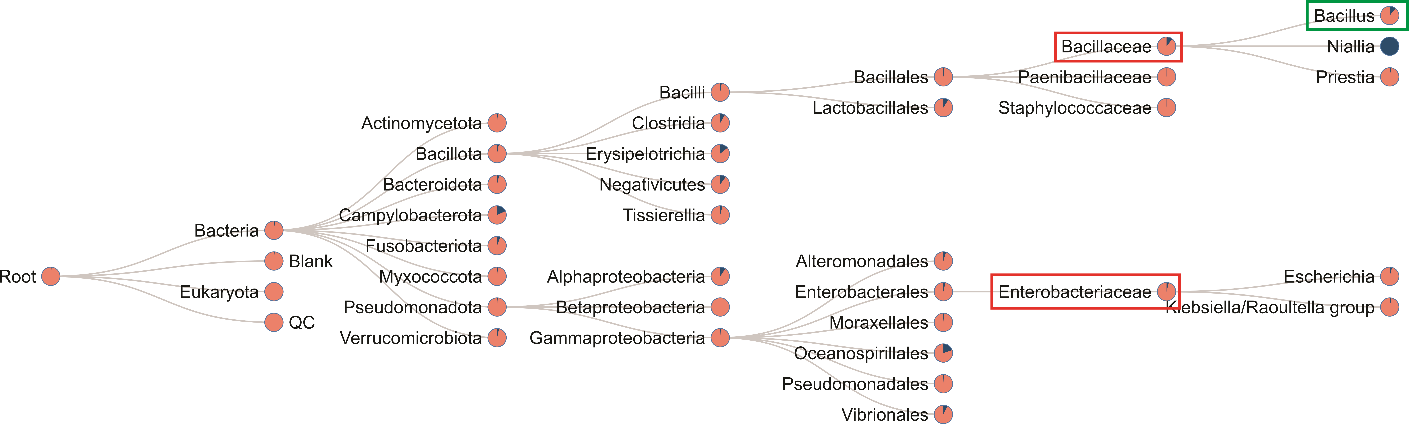


**Figure S3:** Cholic acid spectral search on microbeMASST. Pink nodes represent the presence of the Spectrum USI in different microbial groups.


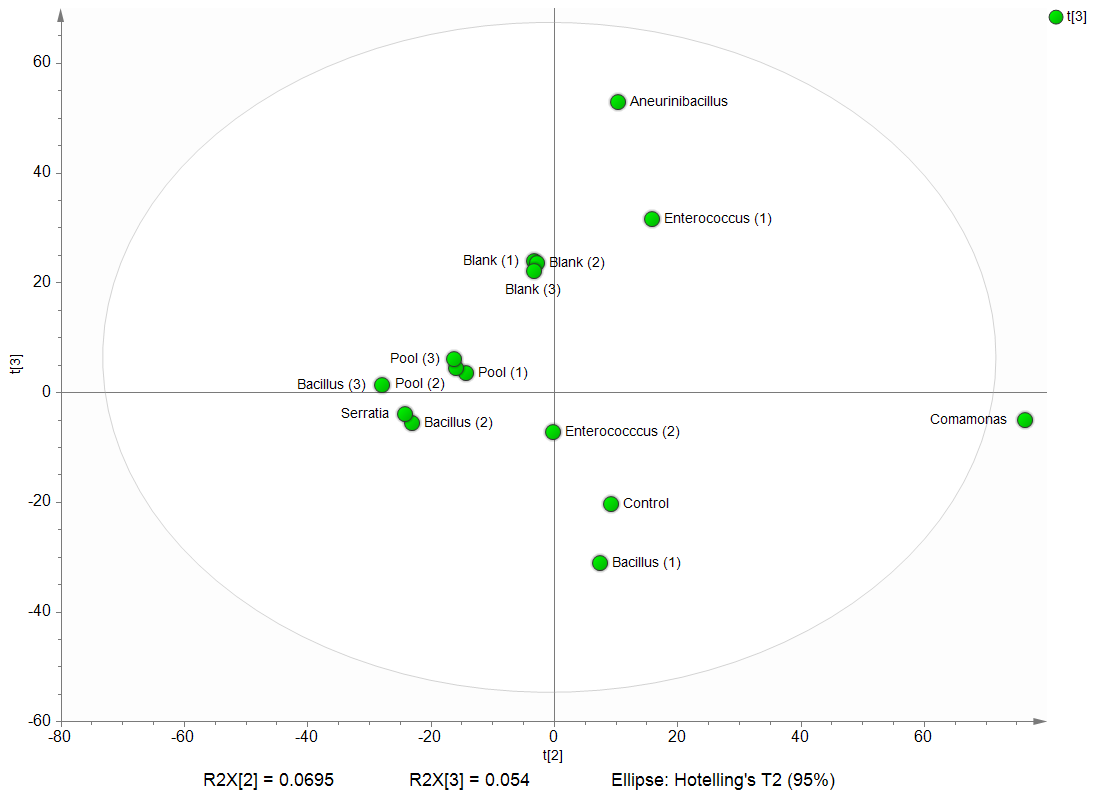


**Figure S4:** Principal Component Analysis (PCA) scores plot of the bacterial samples, blanks, and quality control. The model presented R^2^ of 0.77 and Q^2^ of 0.46 values.

| **Table S2.** Cytotoxic activity rates of eight recovered cave-dwelling bacteria. | | | | | | | | |
| --- | --- | --- | --- | --- | --- | --- | --- | --- |
| **Samples** | **501mel** | | | | **HCT - 116** | | | |
|  | **5µg/mL** | | **50µg/mL** | | **5µg/mL** | | **50µg/mL** | |
|  | **Mean** | **SD** | **Mean** | **SD** | **Mean** | **SD** | **Mean** | **SD** |
| *Bacillus* sp. (1) | 34.426 | 17.86 | 51.072 | 9.881 | 14.532 | 6.924 | 31.448 | 11.694 |
| *Bacillus* sp. (2) | 21.115 | 6.968 | 41.512 | 7.313 | -6.117 | 21.933 | 11.655 | 22.612 |
| *Comamonas* sp. | 42.653 | 21.303 | 68.564 | 6.636 | 13.994 | 17.979 | 38.296 | 5.833 |
| *Serratia* sp. | 21.595 | 18.055 | 39.146 | 11.953 | 5.596 | 8.286 | 20.199 | 6.798 |
| *Enterococcus* sp. (1) | 49.735 | 18.2 | 84.846 | 6.543 | 8.715 | 11.324 | 67.998 | 20.768 |
| *Bacillus* sp. (3) | 13.336 | 13.989 | 27.19 | 8.982 | -5.19 | 8.42 | 1.375 | 16.334 |
| *Aneurinibacillus* sp. | 27.334 | 8.925 | 98.686 | 4.91 | 2.286 | 21.453 | 99.607 | 4.684 |
| *Enterococcus* sp. (2) | 12.178 | 12.44 | 33.496 | 7.332 | 8.348 | 13.188 | 17.037 | 9.89 |

All samples were tested in triplicates.

| **Table S3.** Discriminants features from the PLS-DA, showing the predicted active metabolites. | | | | | | | |
| --- | --- | --- | --- | --- | --- | --- | --- |
| **ID** | ***m/z*** | **RT** | **VIP Score** | **Adduct** | **MS/MS** | **Class** | **Groups** |
| **1155** | 578.373 | 13.55 | 3.27 | (M+2H)^+2^ | 895; 448; 183; 169; 120; 115 | Cyclopeptides | *Comamonas* sp*.; Enterococcus* sp*. (1); Aneurinibacillus* sp. |
| **932** | 426.098 | 11.42 | 3.04 | (M+H)+ | 382; 291; 221; 190; 172; 146 |  | *Comamonas* sp*.; Enterococcus* sp*. (1); Aneurinibacillus* sp. |
| **1429** | 592.367 | 15.80 | 3.02 | (M+2H)^+2^ | 311; 169 | Cyclopeptides | *Comamonas* sp*.; Enterococcus* sp*. (1); Aneurinibacillus* sp*.* |
| **1417** | 585.36 | 15.71 | 2.91 | (M+2H)^+2^ | 339; 169; 120; 115 | Cyclopeptides | *Comamonas* sp*.; Enterococcus* sp*. (1); Aneurinibacillus* sp*.* |
| **613** | 293.129 | 8.09 | 2.85 | (M+2H)^+2^ | 199; 155; 144; 117; 115 | Cyclopeptides | *Comamonas* sp*.; Enterococcus* sp*. (1); Aneurinibacillus* sp*.* |
| **1059** | 564.354 | 12.65 | 2.78 | (M+2H)^+2^ | 434; 169; 155; 120; 115 | Cyclopeptides | *Comamonas* sp*.; Enterococcus* sp*. (1); Aneurinibacillus* sp*.* |
| **1522** | 599.375 | 16.39 | 2.74 | (M+2H)^+2^ | 311; 169; 120; 115 | Cyclopeptides | *Comamonas* sp*.; Enterococcus* sp*. (1); Aneurinibacillus* sp*.* |
| **1508** | 592.367 | 16.30 | 2.65 | (M+2H)^+2^ | 353; 311; 120; 115 | Cyclopeptides | *Comamonas* sp*.; Enterococcus sp. (1); Aneurinibacillus* sp*.* |
| **1562** | 638.38 | 16.61 | 2.55 | (M+2H)^+2^ | 311; 120; 115 | Cyclopeptides | *Comamonas* sp*.; Enterococcus* sp*. (1); Aneurinibacillus* sp*.* |
| **1420** | 1169.71 | 15.73 | 2.54 | (M+2H)^+2^ | 859; 571; 339; 115 |  | *Comamonas* sp*.; Enterococcus* sp*. (1); Aneurinibacillus* sp. |
| **1934** | 647.395 | 18.07 | 2.48 | (M+2H)^+2^ | 169; 120 | Cyclopeptides | *Comamonas* sp*.; Enterococcus* sp*. (1); Aneurinibacillus* sp. |
| **847** | 424.082 | 10.61 | 2.47 | (M+H)^+^ | 372; 354; 219; 172; 146 |  | *Comamonas* sp*.; Enterococcus* sp*. (1); Aneurinibacillus* sp. |
| **37** | 202.09 | 2.54 | 2.44 | (M+H)^+^ | 131 |  | *Bacillus* sp*. (1); Comamonas* sp*.; Enterococcus* sp*. (1); Aneurinibacillus* sp*.; Enterocuccus* sp*. (2)* |
| **212** | 294.113 | 4.55 | 2.43 | (M+H)^+^ | 155; 145 |  | *Comamonas* sp*.; Enterococcus* sp*. (1); Aneurinibacillus* sp*.* |
| **381** | 201.106 | 5.843 | 2.43 | (M+H)^+^ | 131; 125; 107 |  | *Bacillus* sp*. (1); Bacillus* sp*. (2), Comamonas* sp*.; Serratia* sp*.; Enterococcus* sp*. (1); Aneurinibacillus* sp. |
| **862** | 295.059 | 10.80 | 2.41 | (M+H)^+^ | 130 |  | *Comamonas* sp*.; Enterococcus* sp*. (1); Aneurinibacillus* sp*.* |
| **1352** | 585.364 | 15.18 | 2.35 | (M+2H)^+2^ | 339; 311; 169; 120; 115 | Cyclopeptides | *Comamonas* sp*.; Enterococcus* sp. *(1); Aneurinibacillus* sp*.* |
| **1452** | 631.373 | 15.99 | 2.32 | (M+2H)^+2^ | 431; 311; 169; 120; 115 | Cyclopeptides | *Comamonas* sp*.; Enterococcus* sp*. (1); Aneurinibacillus* sp*.* |
| **1876** | 312.253 | 17.78 | 2.30 | (M+2H)^+2^ | 116 |  | *Comamonas* sp.; *Enterococcus* sp. *(1); Aneurinibacillus* sp*.* |
| **1358** | 607.37 | 15.23 | 2.30 | (M+2H)^+2^ | 353; 339; 311; 297; 169; 155; 120; 115 | Cyclopeptides | *Comamonas sp.; Enterococcus sp*. (1); Aneurinibacillus sp. |
| **1497** | 645.371 | 16.27 | 2.29 | (M+2H)^+2^ | 311; 169; 120; 115 | Cyclopeptides | *Comamonas* sp*.; Enterococcus* sp*. (1); Aneurinibacillus* sp*.* |
| **210** | 231.117 | 4.521 | 2.28 | (M+H)^+2^ | 231; 201; 137; 131; 107 |  | *Comamonas* sp*.; Enterococcus* sp. *(1); Aneurinibacillus* sp*.* |
| **1553** | 613.388 | 16.56 | 2.23 | (M+2H^)+2^ | 311; 169; 120; 115 | Cyclopeptides | *Comamonas* sp*.; Enterococcus* sp*. (1); Aneurinibacillus* sp*.* |
| **1626** | 633.379 | 16.91 | 2.18 | (M+2H)^+2^ | 120 | Cyclopeptides | *Comamonas* sp*.; Enterococcus* sp*. (1); Aneurinibacillus* sp*.* |
| **1589** | 583.363 | 16.73 | 2.17 | (M+2H)^+2^ | 569; 534; 526; 120 | Cyclopeptides | *Comamonas* sp*.; Enterococcus* sp*. (1); Aneurinibacillus* sp*.* |
| **1214** | 585.376 | 14.0556 | 2.15 | (M+2H)^+2^ | 325; 183; 120; 115 | Cyclopeptides | *Comamonas* sp*.; Aneurinibacillus* sp. |
| **1341** | 673.396 | 15.09 | 2.10 | (M+2H^)+2^ | 120; 115 | Cyclopeptides | *Comamonas* sp*.; Enterococcus* sp*. (1); Aneurinibacillus* sp*.* |
| **737** | 382.155 | 9.43 | 2.05 | (M+H)^+^ | 250; 182; 134 |  | *Aneurinibacillus* sp. |
